# Supplementary material for: ATR Inhibition Potentiates PARP Inhibitor Cytotoxicity in High Risk Neuroblastoma Cell Lines by Multiple Mechanisms
Source: Cancers (Basel). 2020 Apr 28;12(5):1095. doi: 10.3390/cancers12051095 (PMC7281288; doi:10.3390/cancers12051095)
Supplement: Supplementary file 1 [file cancers-12-01095-s001.pdf]

Supplementary Materials

ATR Inhibition Potentiates PARP Inhibitor Cytotoxicity in High Risk Neuroblastoma Cell Lines by Multiple Mechanisms

Harriet E. D. Southgate, Lindi Chen, Deborah A. Tweddle and Nicola J. Curtin

Supplementary Material

Clonogenic Survival Assay

Exponentially growing cells were seeded into 6 well plates at varying cell densities (50–20,000 cells per well depending on dose of inhibitor) and incubated for 24 h at 37 °C. Drugs were made up at 200× concentration in DMSO before diluting 200× to give 0.5% DMSO. Cells were then treated with VE-821 and/or olaparib for 72 h. Cells incubated in standard media containing 0.5% DMSO were included as controls. After treatment, cells were washed in PBS and incubated for 14–21 days in fresh media until colonies had formed. Colonies were fixed with Carnoy's fixative (methanol/acetic acid, 3:1) and stained with 0.4% (*w*/*v*) crystal violet. Colonies were counted manually. Cloning efficiency (%) ([number of cells seeded/number of colonies counted] ×100) and cell survival (%) ([drug treated cloning efficiency/control cloning efficiency] ×100) were calculated.

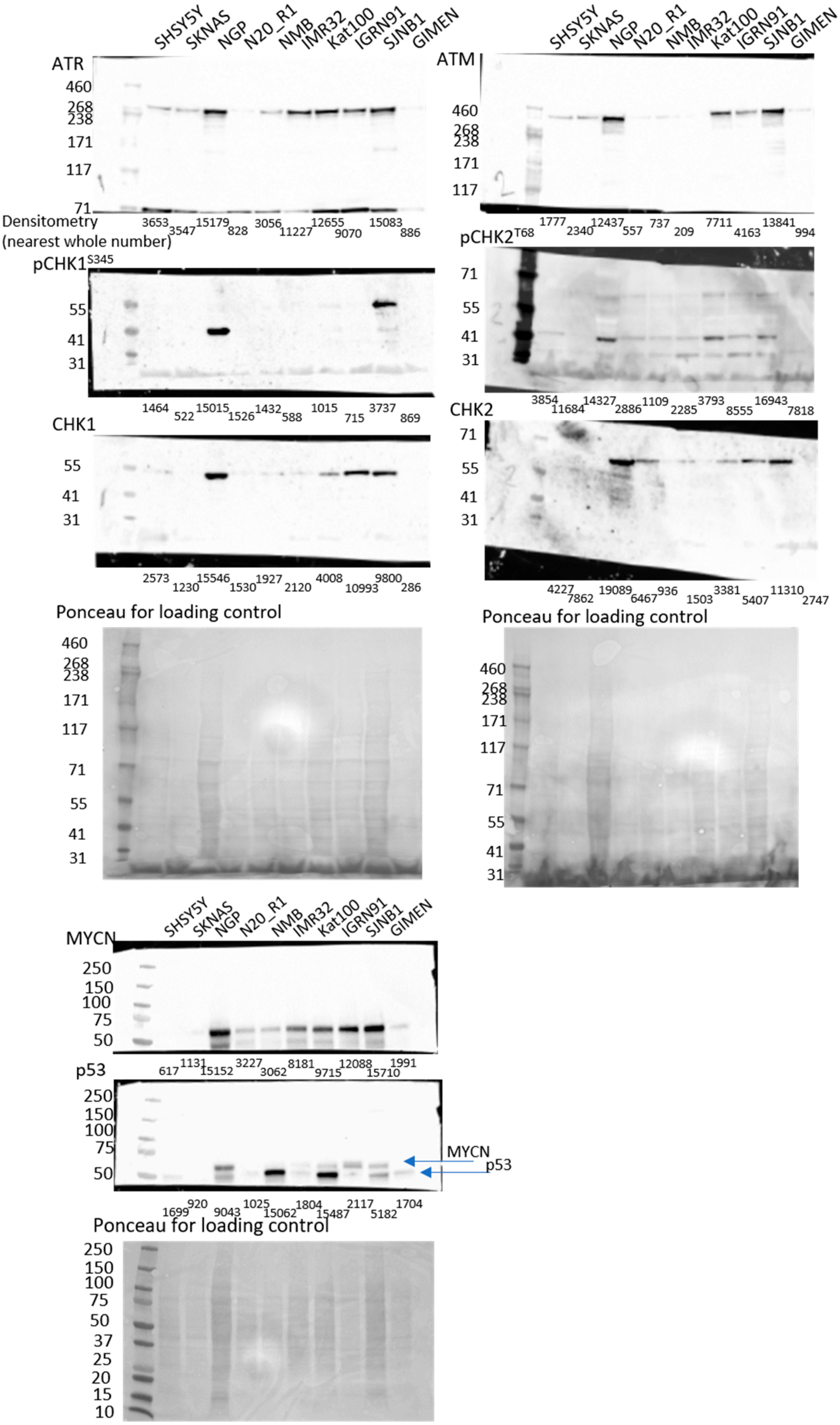

Figure S1. Expression and function of key DDR proteins in a panel of NB cell lines. Full blot images of baseline protein expression of ATR, ATM, CHK1, CHK2, phospho-CHK1^{S345}, phospho-CHK2^{T68} MYCN and p53 in NB cell lines used in this study (Figure 1A).

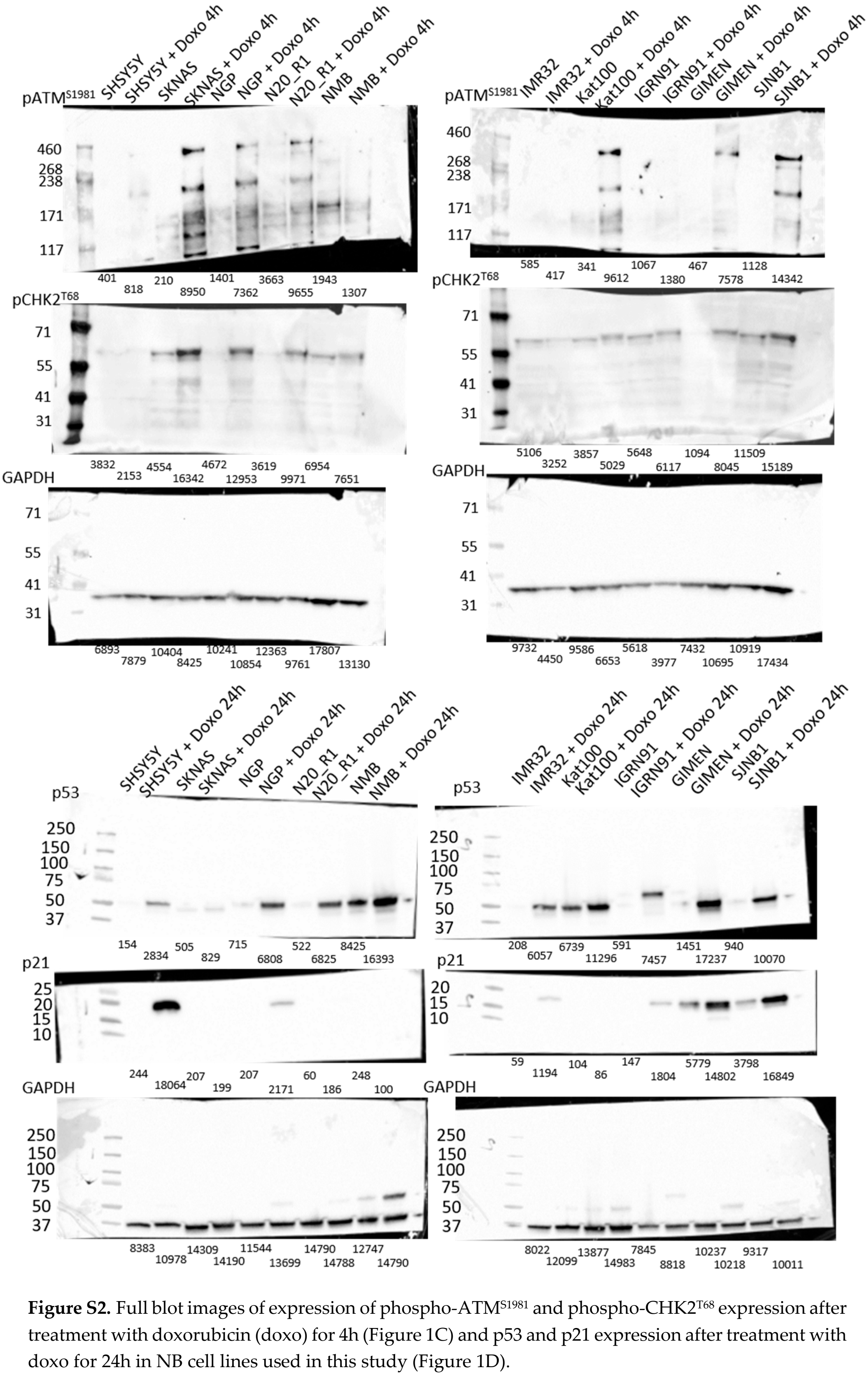

Figure S2. Full blot images of expression of phospho-ATM^{S1981} and phospho-CHK2^{T68} expression after treatment with doxorubicin (doxo) for 4h (Figure 1C) and p53 and p21 expression after treatment with doxo for 24h in NB cell lines used in this study (Figure 1D).

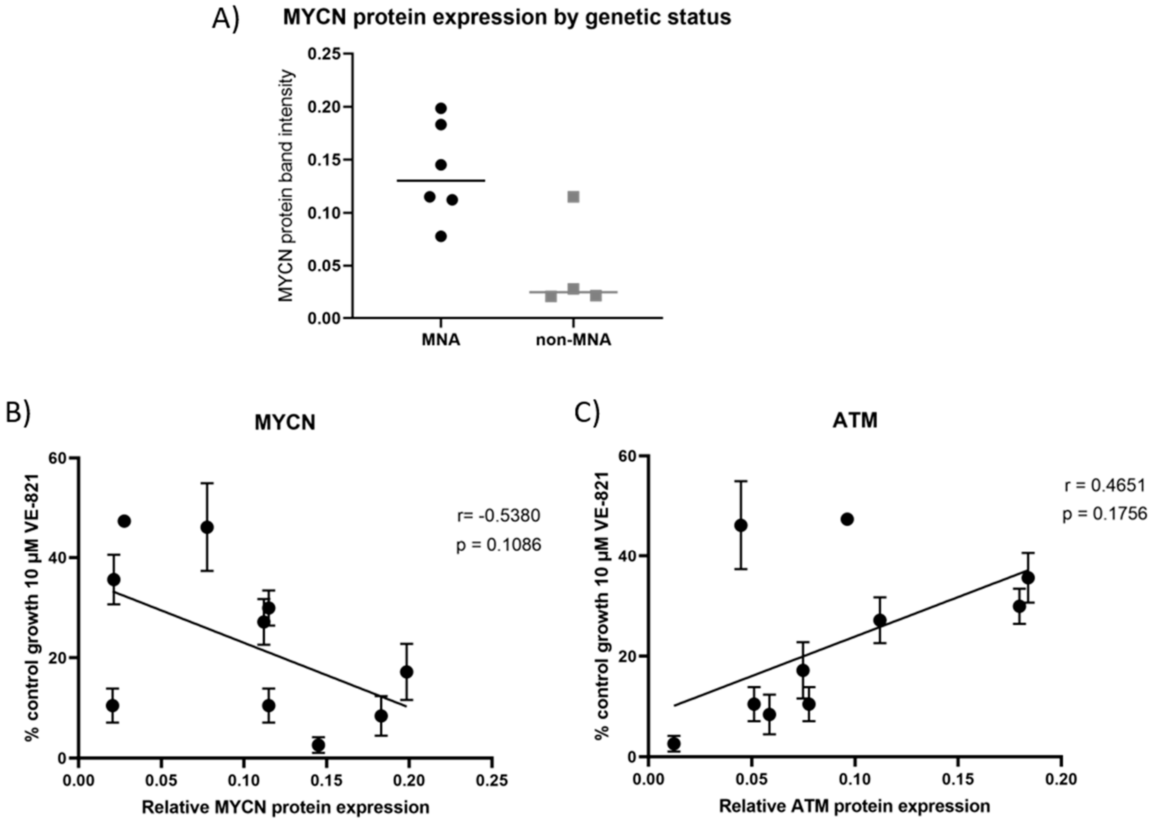

Figure S3. (**A**) Analysis of MYCN protein expression of cell line by *MYCN* amplification status. MNA; MYCN amplified. * $p < 0.05$ Mann Whitney U test. Correlation of relative baseline protein expression of (**B**) MYCN and (**C**) ATM and VE-821 sensitivity at 10 µM. Pearson's rank (r) was tested using Graphpad Prism version 6.

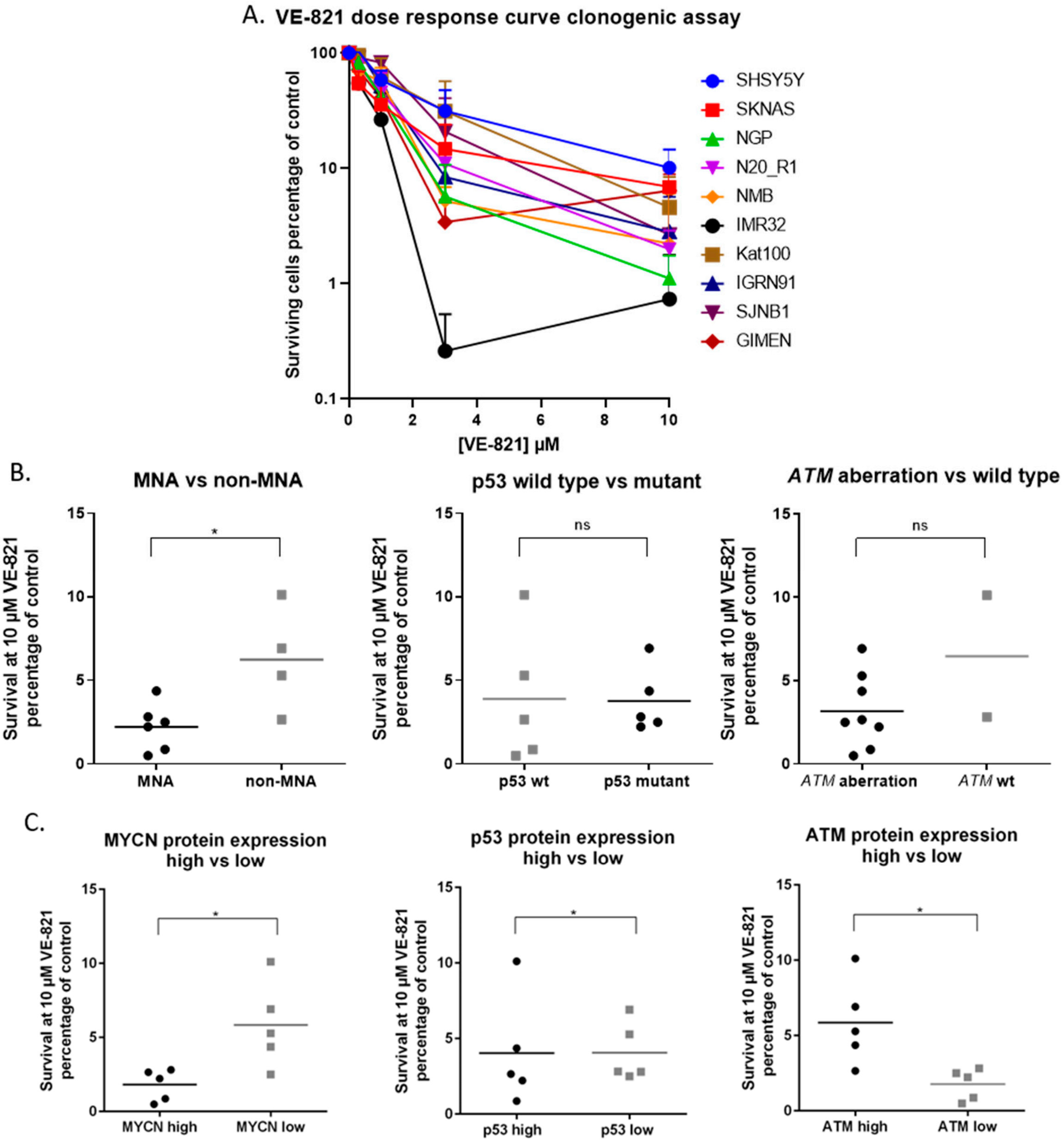

Figure S4. Determinants of ATR inhibitor sensitivity analysis by clonogenic survival assay. (**A**) VE-821 (ATR inhibitor) dose response curves for NB cell lines, data from 3 independent experiments + SEM. Cell lines were split into 2 groups based on (**B**) molecular features, (**C**) protein expression above (high) or below (low) median expression. Average percentage control growth at 10 µM VE-821 was plotted for cell lines belonging to each group ($n = 3$). *$p < 0.05$ Mann Whitney U test, ns: not significant.

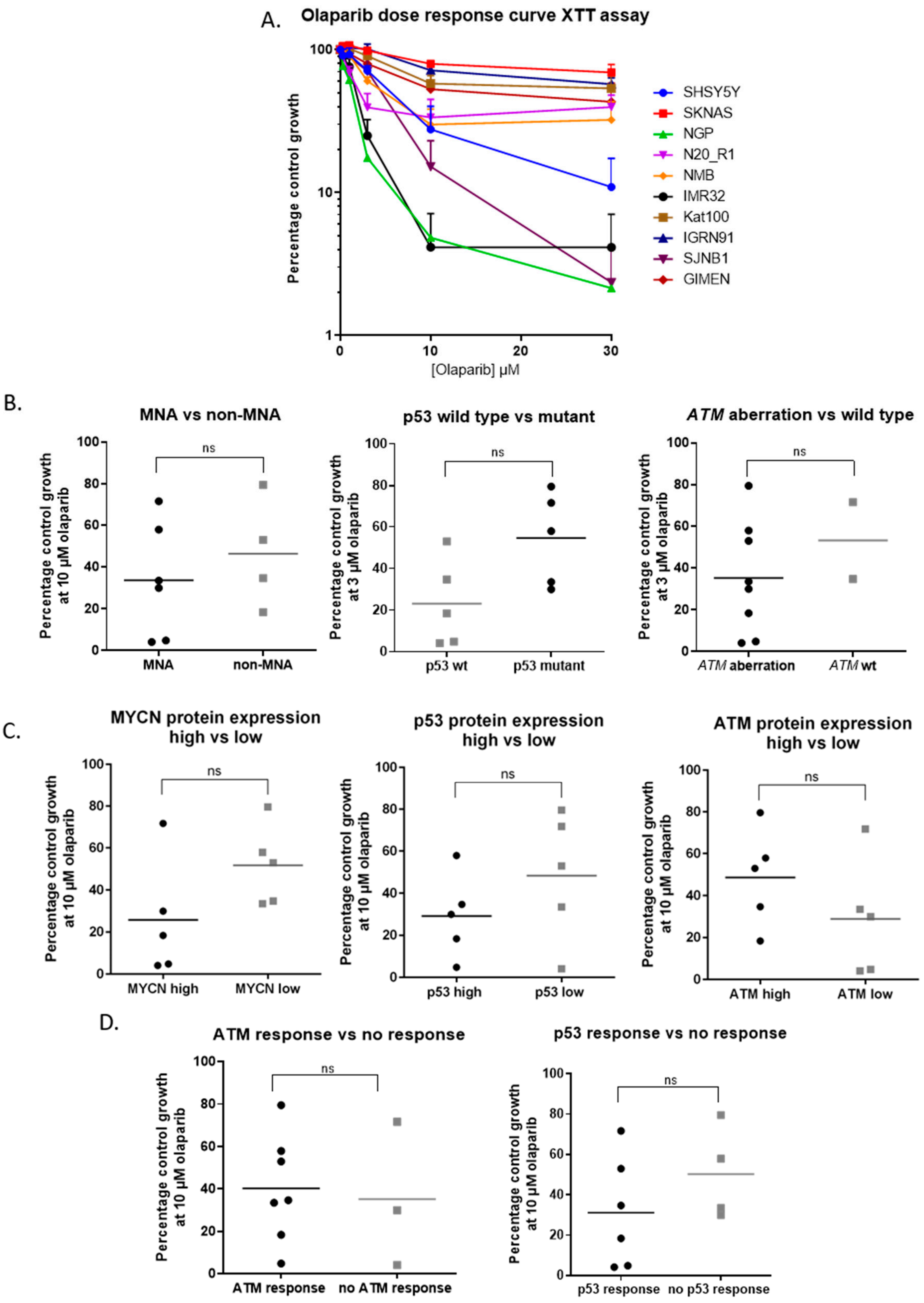

Figure S5. Determinants of PARP inhibitor sensitivity. (**A**) olaparib (PARP inhibitor) dose response curves for NB cell lines, data from 3 independent experiments + SEM. Cell lines were split into 2 groups based on (**B**) molecular features, (**C**) protein expression above (high) or below (low) median expression and (**D**) ATM and p53 responses after treatment with doxorubicin (1 μM). Average percentage control growth at 10 μM olaparib was plotted for cell lines belonging to each group (n = 3). * $p < 0.05$ Mann Whitney U test, ns: not significant.

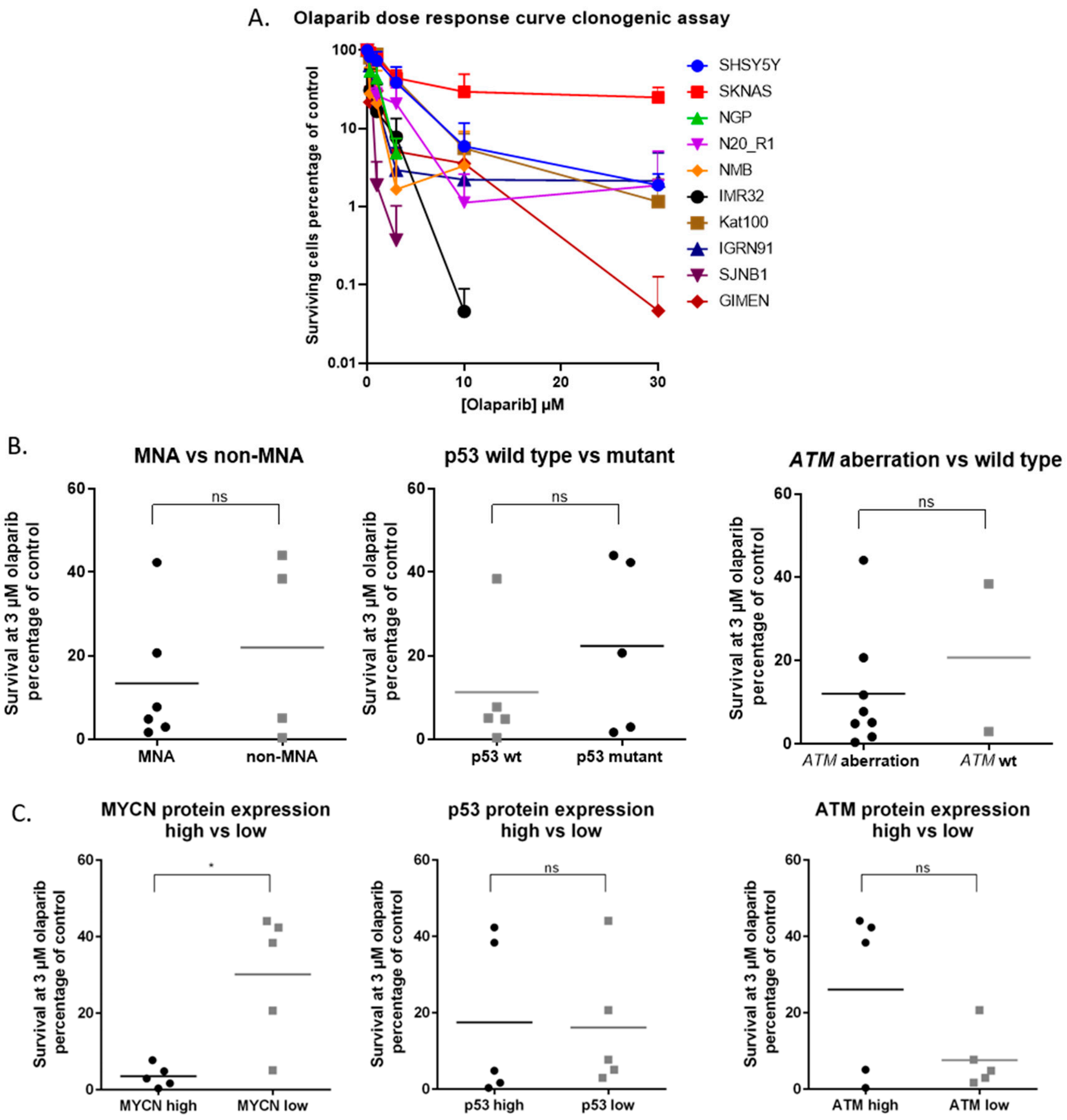

Figure S6. Determinants of PARP inhibitor sensitivity analysis by clonogenic survival assay. (**A**) olaparib (PARP inhibitor) dose response curves for NB cell lines, n = 3 + SEM. Cell lines were split into 2 groups based on (**B**) molecular features, (**C**) protein expression above (high) or below (low) median expression and (**D**) ATM and p53 responses after treatment with doxorubicin (1 µM). Average percentage control growth at 10 µM olaparib was plotted for cell lines belonging to each group (n = 3). * $p < 0.05$ Mann Whitney U test, ns: not significant.

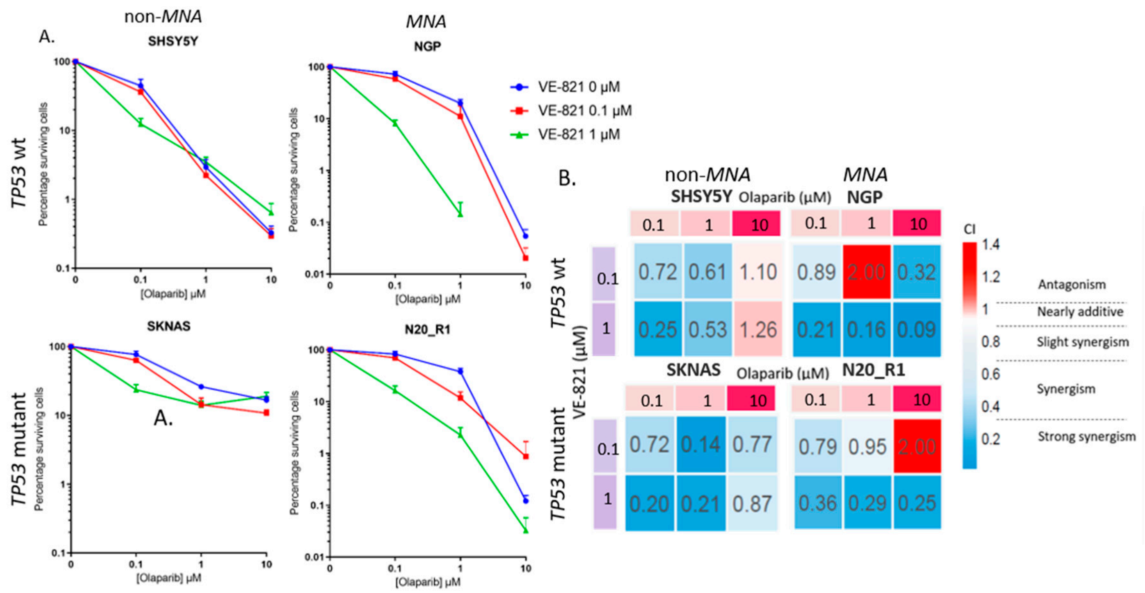

Figure S7. (**A**) clonogenic survival assay of the SHSY5Y, SKNAS, NGP and N20_R1 neuroblastoma cell lines treated with 0.1, 1 and 10 µM olaparib alone and with the addition of 0.1 and 1 µM VE-821. Percentage survival was normalised to effect of VE-821 alone, data from 3 independent experiments + SEM. (**B**) Heat map of combination index (CI) values were calculated using CalcuSyn.

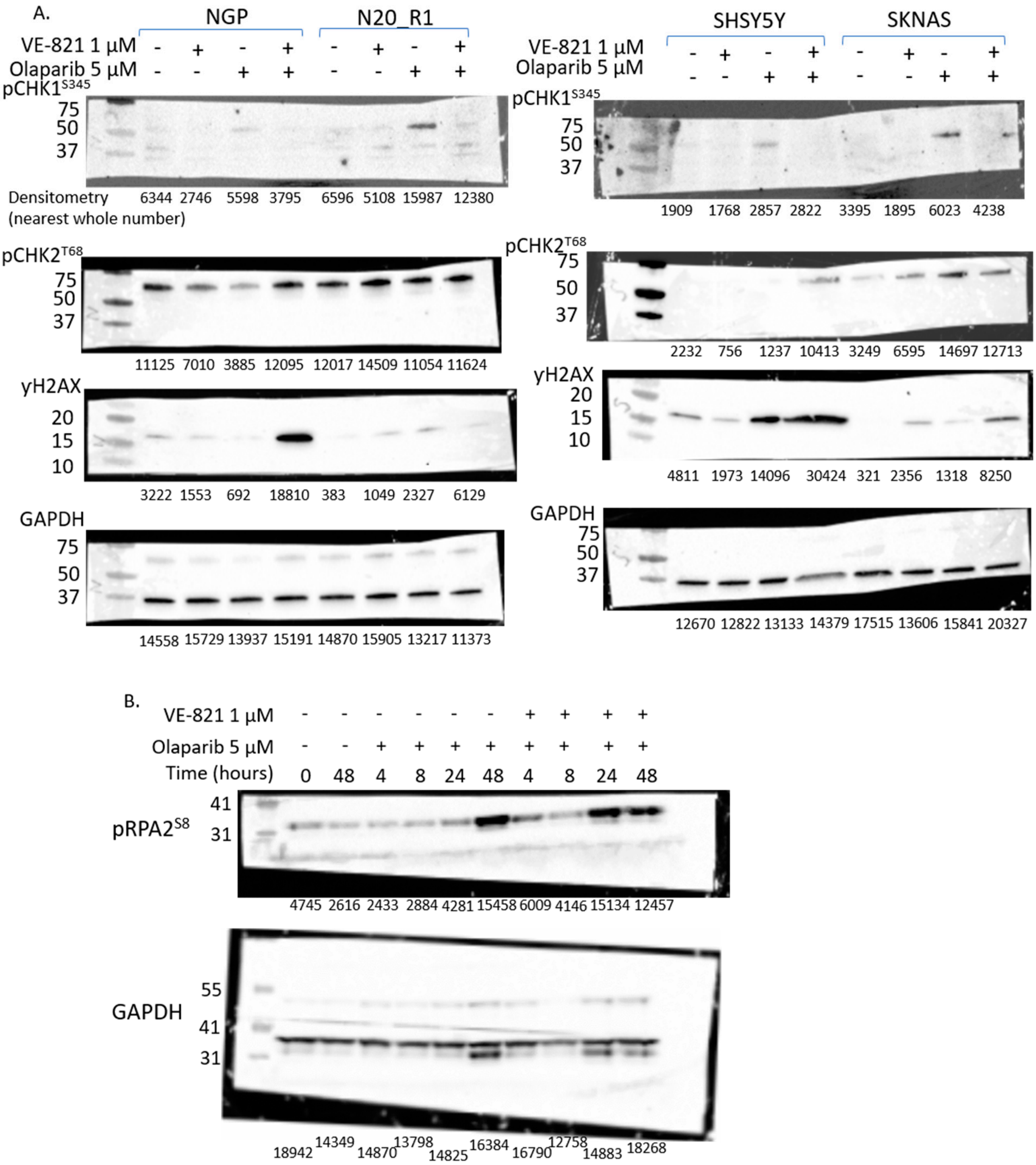

Figure S8. Effect of VE-821 and olaparib combination on replication stress. (**A**) Full blot images pChk1^{S345}, pChk2^{T68}, γH2AX protein expression of NGP, N20_R1, SHSY5Y and SKNAS cells after incubation with 5 µM olaparib and/or 1 µM VE-821 for 24 hours (Figure 4A). (**B**) Full blot images of pRPA2^{S8} expression in SHSY5Y cell line in response to 5 µM olaparib with or without 1 µM VE-821 over 48 hours (Figure 4C).

© 2020 by the authors. Licensee MDPI, Basel, Switzerland. This article is an open access article distributed under the terms and conditions of the Creative Commons Attribution (CC BY) license (http://creativecommons.org/licenses/by/4.0/).
